# A study of nonuniform CTV to PTV margin expansion incorporating both rotational and translational uncertainties

**DOI:** 10.1002/acm2.12763

**Published:** 2019-12-02

**Authors:** Junjie Miao, Yingjie Xu, Yuan Tian, Zhiqiang Liu, Jianrong Dai

**Affiliations:** ^1^ Department of Radiation Oncology National Cancer Center/National Clinical Research Center for Cancer/Cancer Hospital Chinese Academy of Medical Sciences and Peking Union Medical College Beijing China

**Keywords:** nonuniform, rotational error, setup uncertainty, target margin

## Abstract

**Purpose:**

In this work, we implemented a method to obtain a nonuniform clinical target volume (CTV) to planning target volume (PTV) margin caused by both rotational and translational uncertainties and evaluated it in the treatment planning system (TPS).

**Materials and method:**

Based on a previously published statistical model, the relationship between a target margin and the distance *d* (from isocenter to target point), setup uncertainties, and significance level was established. For a single CTV, it can be thought as a combination of many small volume elements or target points. The margin of each point could be obtained using the suggested statistical model. The whole nonuniform CTV–PTV margin was determined by the union of all possible margins of the CTV boundary points. This method was implemented in the Pinnacle^3^ treatment planning system and compared with uniform margin algorithm. Ten vertebral metastases targets and multiple brain metastases targets were chosen for evaluation.

**Results:**

The combined CTV–PTV margin as a function of *d* for various initial translational margin and rotational uncertainties was calculated. The combined margin increases as *d*, rotational uncertainties and translational margin increase. For the same rotational uncertainty, a smaller initial translational margin requires a larger rotational margin to compensate for the rotational error. Compared with the uniform margin algorithm, the advantage of this method is that it could minimize the PTVs volume for given CTVs to obtain same significance level. Using vertebral metastases targets and multiple brain metastases targets, a series of volume difference was obtained for various translational margins and rotational uncertainties. The volume difference of PTV could be more than 17% when translational margin is 2 mm and rotational uncertainty is 1.4°.

**Conclusion:**

Nonuniform margin algorithm could avoid excessive compensation for the CTV boundary points near isocenter. This method could be used for clinical margin determination and might be useful for the protection of risk organs.

## 1 INTRODUCTION

It is well known that setup uncertainties would be introduced throughout the treatment delivery. These setup errors during treatment are critical to the success of radiotherapy.^1^, ^2^, ^3^, ^4^ Without proper attention, these setup errors can cause misalignment of the beams and lead to the radiation dose delivered outside the target area. To solve this problem, a method is provided to account for setup errors in the ICRU 50 report and its supplement.^5^ According to ICRU 50, a margin is added to the clinical target volume (CTV), thus yielding the planning target volume (PTV). The entire PTV is given a prescription dose to ensure that the CTV receives desired coverage. During the past years, many recipes for CTV–PTV margin have been developed by different groups.^6^, ^7^, ^8^, ^9^, ^10^, ^11^

Usually, PTV is formed by drawing a uniform margin around CTV to account for setup errors and possible motion during the treatment. The setup errors include both translational and rotational setup uncertainties. Currently, kilovoltage cone beam computed tomography (kV‐CBCT) and Optical Surface Monitoring System (OSMS) are widely used in clinical practice to provide image guidance for treatment.^9^, ^12^ Both three‐dimensional translational and rotational positioning errors can be detected and measured. In current clinical practice, translational errors can be corrected online since they are easily implemented using couch shifts along three axes. Rotational setup differences between the patient's position in the linac and the CT scanning position can be corrected using advanced couches that have six degrees of freedom.^13^, ^14^ While the use of such couches is increasingly prevalent, there are still many linacs with conventional couches that cannot correct for pitch, roll, or yaw. These common linear accelerators were often installed earlier or in the underdeveloped areas. For the rotational errors in these common linear accelerators, some correction algorithms or methods need to be used.

Many studies on PTV computation methods only focus on translational uncertainties. For some situations, however, rotational uncertainties play an important role. For example, Sasaki et al. and Liu et al. studied dosimetric impact of translational and rotational setup errors for spine and lung stereotactic body radiotherapy. They found even if the rotational setup error was ≤2°, rotational errors alone could cause an unexpected dosimetric effect. They found the rotational setup error was related to the isocenter location, and rotational setup error would be more significant if the isocenter is far from the geometric center of the target. To account for these rotation errors, an extra margin is needed for the margin between CTV and PTV.^15^, ^16^ Zhang et al. reported an analytical formula to determine the extra margin between CTV and PTV to account for both translational and rotational setup errors.^17^ Remeijer et al. described a probability‐based approach to generate margins for translational and rotational uncertainties.^18^ Most of the previous studies usually use uniform CTV–PTV margin which may lead to a large PTV.

In addition, a new technology called single‐isocenter for multiple target (SIMT) technique has been developed in recent years. In SIMT technique, one isocenter which is usually located in the geometrical center of the total CTVs is used for treating multiple lesions.^19^ With SIMT technique, significant time can be saved for patient setup and radiation delivery. But this technique will also introduce additional rotational uncertainties and these uncertainties are not easy to be corrected with IGRT and six‐degree couch for all targets. The additional rotational uncertainty could not be ignored when the distance between isocenter and target is large. Roper et al. reported that target coverage was significantly lower than expected when rotational errors (>1°) were introduced.^20^ Chang developed a new margin recipe using a statistical model to investigate the effect of additional rotational error for the SIMT technique.^21^ But the margin size for single target in one direction (x, y, or z) is also uniform.

The size of extra CTV–PTV margin introduced by rotational uncertainties depends on the treatment site, the distance from the isocenter to the target, rotational uncertainties, and the required confidence level for tumor coverage.^10^ If isocenter, rotational uncertainties, and the required confidence level have already been fixed, the extra margin size of one boundary point in target is mainly related to the distance between the point and the isocenter. So the needed extra margin size caused by rotation will change when the CTV points are in different boundary location. If a uniform value is chosen as the whole target margin, which is usually close to the maximum of all points, it would cause the target volume to be very large. As a result, large target volume will increase the dose of the surrounding organs.

In this paper, we propose a method to obtain a nonuniform CTV–PTV margin caused by setup uncertainties. This method is based on a statistical model considering both the conventional translational error and the additional rotational uncertainty. The benefits of this method will be investigated by comparing it with uniform margin algorithms. For rotational uncertainties of all points at the CTV boundary are considered, the CTV–PTV margin recipe derived from this method will better reflect the expansion necessary. This method could be used for clinical margin determination and might be useful for the protection of organs nearby.

## 2 MATERIALS AND METHODS

### 2.1 The statistical model

**Figure 1:**
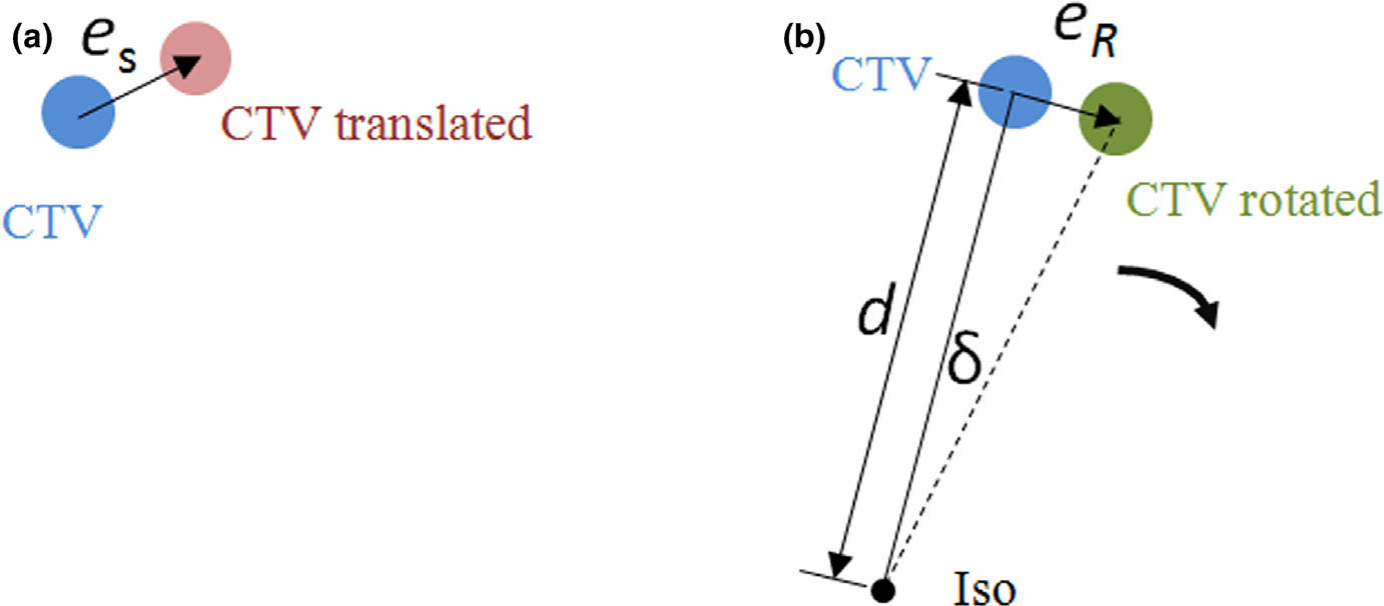
Illustration of the (a) translational and (b) rotational errors. The translational error in (a) is a random vector. The amplitude and direction remain the same regardless of the location of the clinical target volume (CTV). The amplitude of rotational error in (b) increases with d, the distance between the isocenter and the CTV.

Figure 1 illustrates the translational and rotational errors that introduce uncertainty to the CTV location. For simplicity and convenience, it is assumed that the axis of rotation goes the isocenter. The translational error vector ***e*** *_S_* as shown in Fig. 1(a) is a random vector with fixed amplitude and direction regardless the CTV location. In Fig. 1(b), the CTV rotates around the axis that is normal to the paper passing through the isocenter (ISO) for *δ* degrees. The rotational error ***e*** *_R_* is also a random vector. However, its amplitude is equal to sin*δ* × *d* ≈ *δd* (for small *δ*), where *d* is the distance between the isocenter and the CTV point. And its direction is not fixed but along the rotational direction. When considering the CTV to PTV expansion, the combined setup error ***e***_R_ + ***e***_S_ (translated & rotated) needs to be compensated.

Chang has developed a statistical model to analyze the rotational error for the SIMT technique.^21^ In this model, the translational setup error follows the three‐dimensional independent normal distribution with zero mean and a standard deviation (SD) of ***σ***_s_ (in mm). It is assumed that the rotation happens randomly and follows a three‐dimensional (3D) independent normal distribution with a zero mean and a uniform SD of ***σ***_D_ (in degrees). Correspondingly, rotational setup error determined by rotation also follows a 3D‐independent normal distribution with a zero mean and a uniform SD of $\sigma_{R}=0.816d\sigma_{D}\frac{\pi }{180}=0.01424d\sigma_{D}$ (in mm). The value of *σ_R_* is proportional to *d* (the distance between the isocenter and the CTV point) and rotational uncertainty *σ*_D_. Based on the published method,^22^ the combined PTV margin is

(1) $$\begin{array}{cc} M_{E}=\chi_{\alpha }\sigma_{E}= & \chi_{\alpha }\sqrt{\sigma_{S}^{2}+\sigma_{R}^{2}}\\ = & \chi_{\alpha }\sqrt{\sigma_{S}^{2}+{\left(0.01424d\sigma_{D}\right)}^{2}}\\ = & \sqrt{M_{S}^{2}+M_{R}^{2}}. \end{array}$$

where $\chi_{\alpha }^{2}$ is the critical value of Chi‐square distribution with three degrees of freedom for significance level *α*. And $\chi_{\alpha }$ values for several probability levels are given in Table 1. $M_{S}=\chi_{\alpha }\sigma_{S}$ is the required PTV margin for the translational error, and $M_{R}=\chi_{\alpha }\sigma_{R}$ is the required PTV margin for the rotational error. From Eq. (1), it is clear that the combined PTV margin *M_E_* is related with translational margin *M*_S_, rotational uncertainty *σ*_D_, and the distance *d* (between the isocenter and the CTV point).

**Table 1:** Chi‐square distribution table with three degrees of freedom, $\chi_{\alpha }^{}$ is the critical value of Chi‐square distribution, and *α* is significance level.

1 − α	χ_α_	1 − α	χ_α_
0.9	2.5	0.95	2.795
0.91	2.548	0.96	2.883
0.92	2.6	0.97	2.991
0.93	2.657	0.98	3.136
0.94	2.722	0.99	3.368

### 2.2 Nonuniform margin

**Figure 2:**
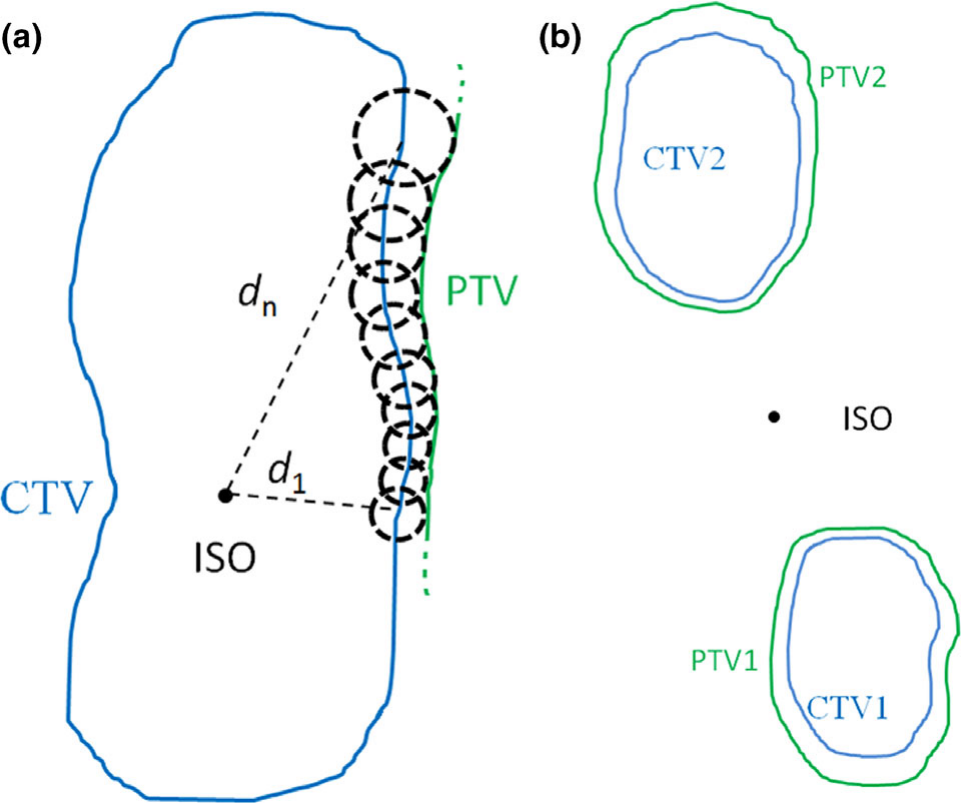
Illustration of a method to obtain a nonuniform clinical target volume (CTV)–planning target volume (PTV) margin. The PTV is constructed by the union of all possible displacements of the CTV boundary points. (a) For a single irregular target with isocenter in the CTV. (b) For the SIMT technique, the isocenter is outside the target area.

For a single target, it can be thought as a combination of many small volume elements or target points. Based on the above statistical model, the margin of every point could be obtained using Eq. (1). The whole CTV–PTV margin is determined by all possible margins of the CTV boundary points. In this paper, $\chi_{\alpha }$ value, translational setup error, and rotational setup angle of boundary points are assumed to be fixed value. Since the distance between isocenter and every CTV boundary point is different, the margin of each CTV boundary point will not be equal. Finally, we would obtain PTV as the union of CTV nonuniform expansion as shown in Fig. 2(a). For the SIMT technique, the isocenter is usually outside the target area, and the nonuniform margin for every target will be calculated one by one as shown in Fig. 2(b).

### 2.3 Implementation of the method

**Figure 3:**
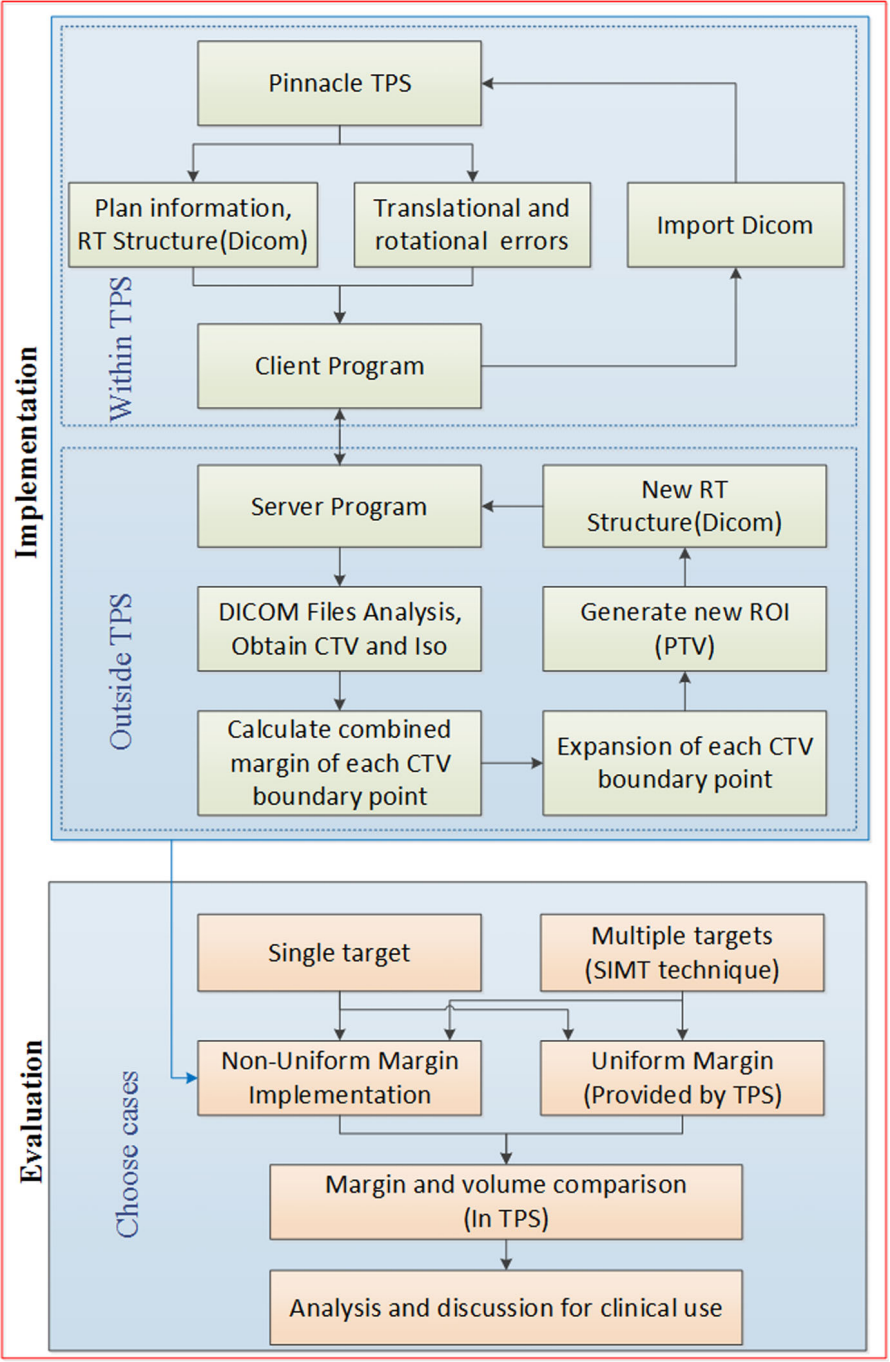
Flowchart of the procedures in this work.

The method was implemented in the Pinnacle^3^ treatment planning system (version 9.10, Philips). The gross tumor volume (GTV) and CTV were delineated and reviewed by experienced radiation oncologists based on the planning CT. Pinnacle^3^ Scripts and custom Python code were used to export CTV contour, ISO location and other position information into DICOM files, which includes RT‐Structure and RT‐Plan. Then parameters of contoured structures were extracted from the DICOM files using the software package Computational Environment for Radiotherapy Research (CERR).^23^ The contoured structures were converted into 3D matrix using an open‐source software visualization toolkit (VTK)‐based algorithm. The matrix dimension was same as CT images, which were 512 × 512 pixels in transverse plane and 3‐mm slice thickness in axial direction. CTV in 3D matrix format was processed with custom Python code to obtain a nonuniform margin. At last, the PTV in DICOM format was imported into Pinnacle^3 ^treatment planning system by Pinnacle^3^ Scripts. A flow diagram depicting this process could be seen in Fig. 3.

### 2.4 Evaluation of the proposed method

This method was compared with uniform margin algorithm provided by Pinnacle, which was widely used now. We chose vertebral metastases targets and multiple brain metastases targets to test this method because relative motion between different parts of the targets exist, and the uncertainties are not easy to be corrected as desired using six‐degree couch with CBCT. Ten patients with vertebral metastases targets were chosen and the isocenter of each plan was located on the geometric center of the CTV. The significance level was set to be 1 − α = 0.95, then the corresponding $\chi_{\alpha }$ would be equal to 2.795. In the uniform margin algorithm, the margin of the CTV was chosen to make the same significance level. For translational margin (M_S_ = 2 mm) and a series of *σ*_D_ (0.4°, 0.6°, 0.8°,1.0°, 1.2°, 1.4°) values, the volumes obtained by uniform margin algorithm (V_uniform_) and nonuniform margin algorithm (V_non‐uniform_) were compared.

Based upon this model, we chose ten patients with multiple brain metastases targets treated with the SIMT technique. The translational margin is set to be 2 mm, and $\chi_{\alpha }$ is 2.795 (corresponding significance factor is 0.95). The isocenter was chosen as the geometrical center of the combined CTVs (CTV1 + CTV2), which is outside all CTVs. The range of CTV‐PTV margin and volumes with a series of rotational uncertainties were calculated and compared in Pinnacle. A flowchart depicting whole processes of the study is shown in Fig. 3.

## 3 RESULTS

### 3.1 Variety of margin with distance

**Figure 4:**
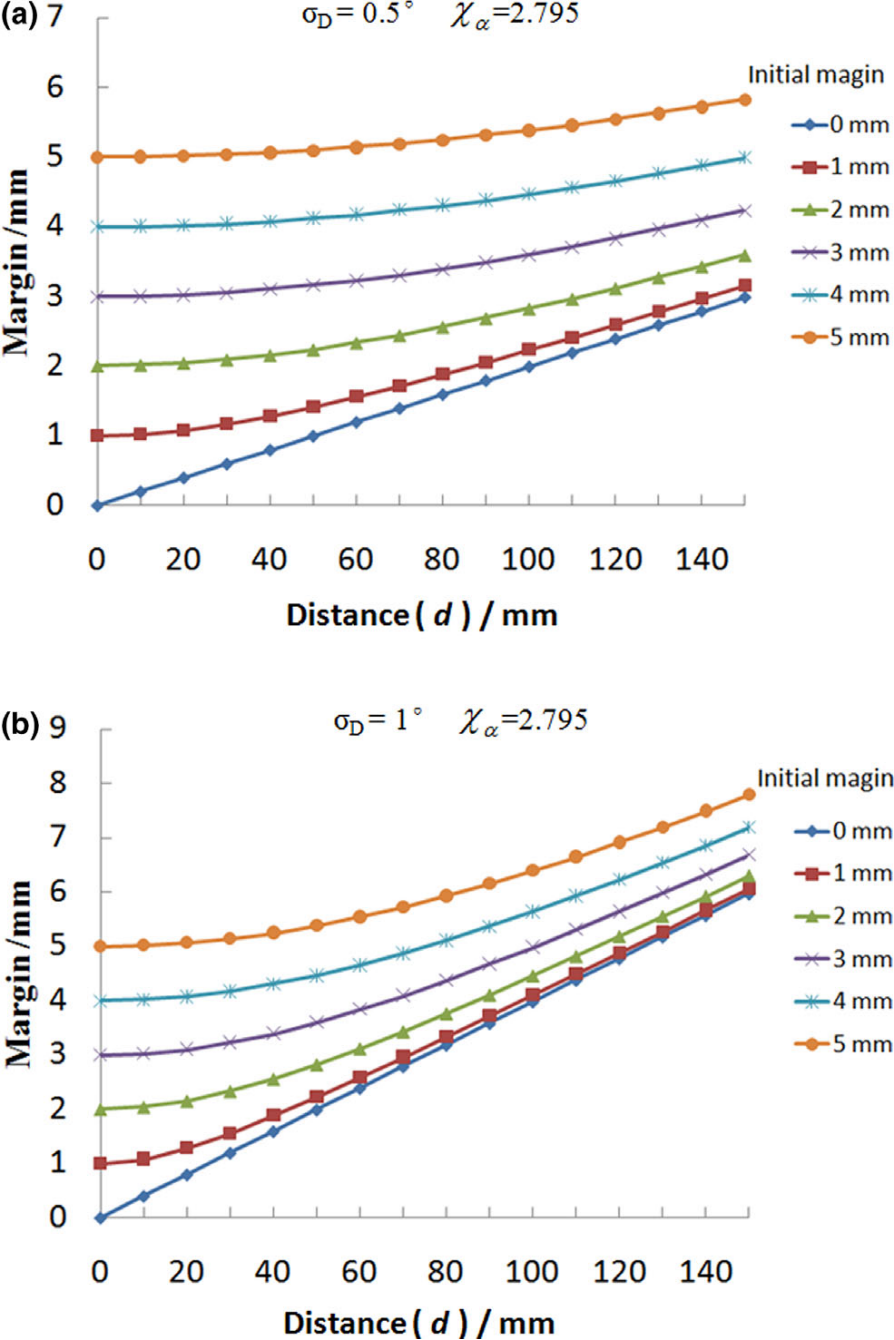
The combined clinical target volume (CTV)–planning target volume margin as a function of *d* (the distance between the isocenter and the CTV point) for various initial translational margin *M*_S_ to achieve the same coverage probability. The range of *d* is from 0 to 150 mm with 10 mm increment, in which the parameter sets are $\chi_{\alpha }$ = 2.795, *σ*_D_ = 0.5° for (a) and *σ*_D_ = 1.0° for (b).

Figure 4 provides the combined CTV–PTV margin as a function of *d* (the distance between the isocenter and the CTV point) for various initial translational margin *M*_S_ to achieve the same coverage probability, that is, 95% of the time the prescription dose would cover the CTV point. Figure 4 (a) plots the combined margin versus *d* ranging from 0 to 150 mm with 10 mm increment, in which the parameter sets are *σ*_D_ = 0.5°and $\chi_{\alpha }$ = 2.795. Figure 4(b) is the plot for *σ*_D_ = 1.0° case with other settings similar with Fig. 4(a). In these two figures, they both contain six plots with initial translational margin *M*_S_ ranging from 0 to 5.0 mm with 1 mm increment. It is clear from Fig. 4 that the combined CTV–PTV margin increases as *d* and translational margin increase.

**Figure 5:**
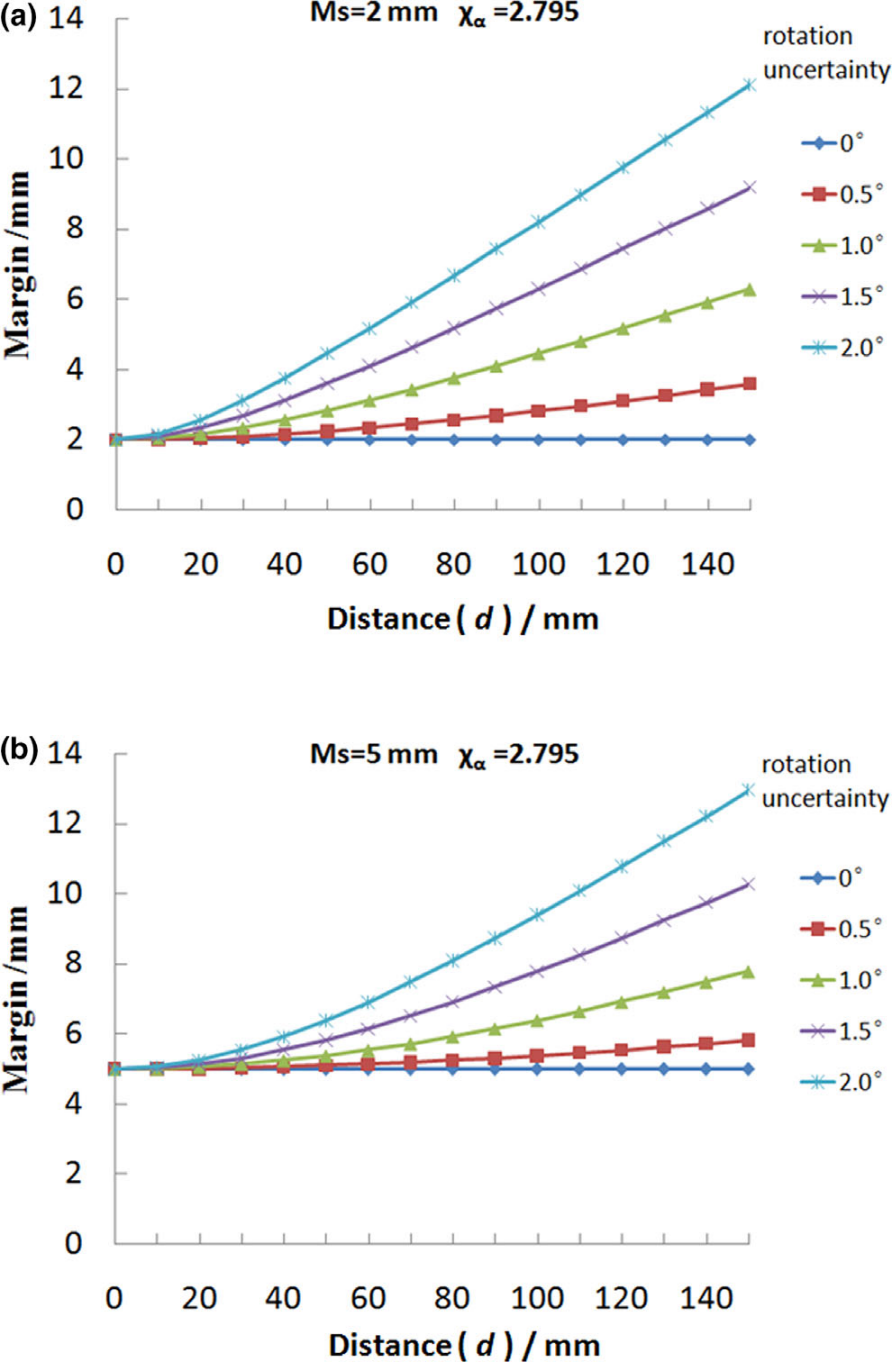
The combined clinical target volume (CTV)–planning target volume margin as a function of *d* for various rotational uncertainties to achieve the same coverage probability, that is, 95% of the time the prescription dose would cover the CTV point. The range of *d* is from 0 mm to 150 mm with 10 mm increment, in which the parameter sets are $\chi_{\alpha }$ = 2.795, *M* ***_S_*** **= 2 mm for (a) and *M* ***_S_*** = 5 mm for (b).

The margins for translational error are usually about 5, 2 mm for normal fractionated technique and SBRT respectively. In Fig. 5, the combined CTV–PTV margin as a function of *d* for various rotational uncertainties is shown. The range of *d* is from 0 to 150 mm with 10 mm increment, in which the parameter sets are $\chi_{\alpha }$ = 2.795, *M* ***_S_*** **= 2 mm for (a) and *M* ***_S_*** = 5 mm for (b). As shown in Fig. 5, it is clear that the combined margin would be a fixed value with variable *d* when the rotational uncertainty was 0°. For other nonzero rotational uncertainties, the combined CTV–PTV margin is positively related to variable *d*.

### 3.2 Vertebral metastases

**Figure 6:**
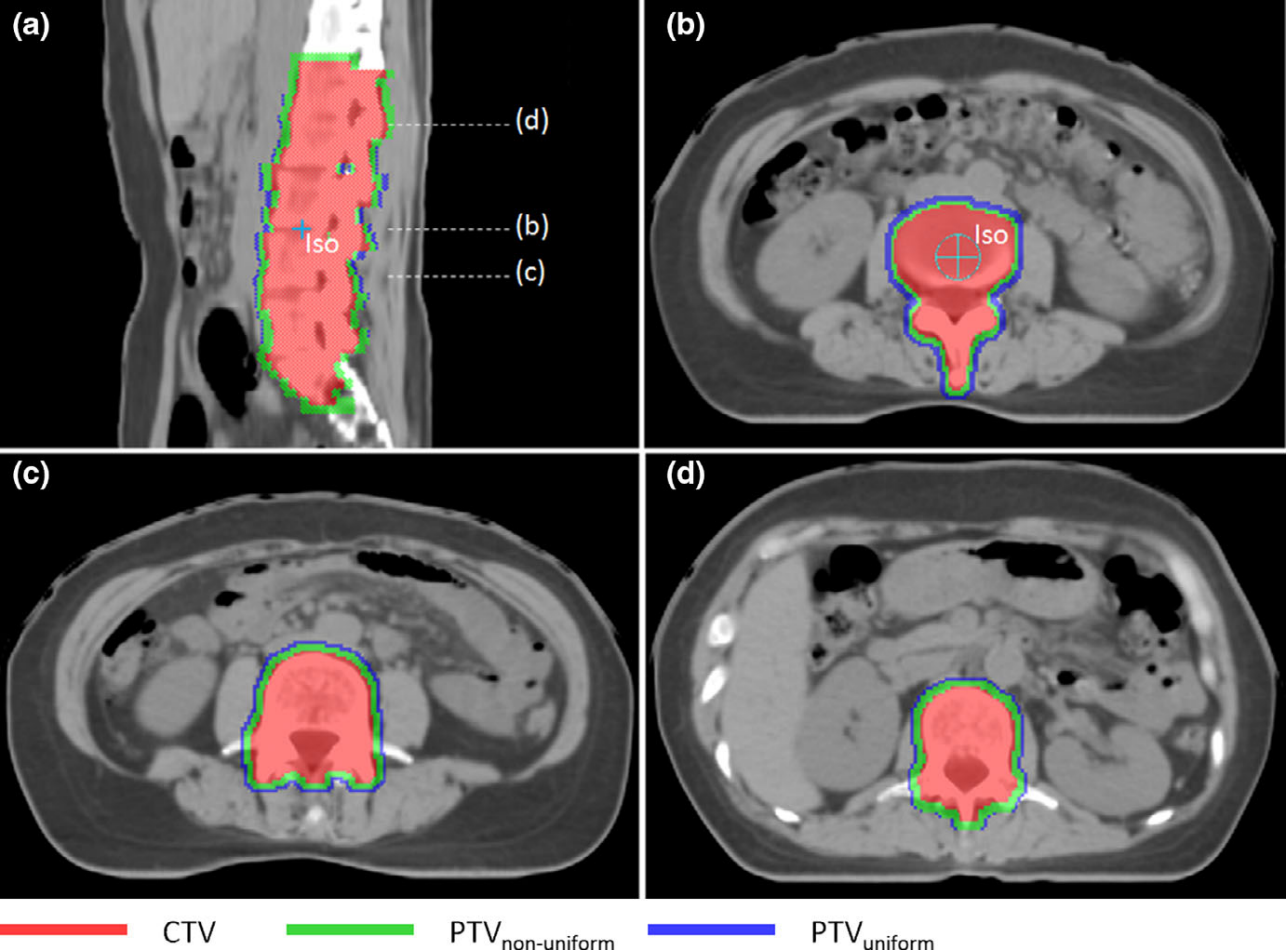
A comparison of planning target volume generated via nonuniform (green) algorithm and uniform (blue) algorithm for a vertebral metastases targets. (a) In sagittal view. (b), (c) and (d) are transverse views of the patient with different distances from isocenter.

Figure 6 shows the comparison of PTV generated via nonuniform (green) algorithm and uniform (blue) algorithm for a vertebral metastases target. Figures 6(b)–6(d) are transverse views of the patient with different distance from isocenter. It is clear that the largest difference of PTV_uniform_ and PTV_non‐uniform_ is at the transverse section with isocenter. The difference of PTV_uniform_ and PTV_non‐uniform_ becomes smaller and smaller when the transverse section is close to the ends of the target. Table 2 shows the results of volume difference between V_uniform_ and V_non‐uniform_ with a series of M_S_, *σ*_D_ values. From the volume comparison, we can see that the PTV volume obtained by our nonuniform margin algorithm is less than volume obtained by the uniform margin algorithm. When the rotational uncertainty is small, volume difference is almost negligible. But if this rotational uncertainty becomes big, the volume difference of the PTV will be significant. The maximum value of volume difference in Table 2 is more than 17%, and the corresponding *M*_S_, and *σ*_D_ values are 2 mm, and 1.4°, respectively. It can be easily predicted that this volume difference will become bigger if the values *M*_S_ and *σ*_D_ further increase.

**Table 2:** The results of volume difference between V_uniform_ and V_non‐uniform_ for ten vertebral metastases with a series of M_S_, $\sigma_{D}$($\chi_{\alpha }$ = 2.795). V_CTV_ and L_CTV_ are the volume and length of clinical target volume, respectively.

Patient	V_CTV_ (cm^3^)	L_CTV_ (cm)	Volume difference (%)					
			2 mm 0.4°	2 mm 0.6°	2 mm 0.8°	2 mm 1.0°	2 mm 1.2°	2 mm 1.4°
1	358.7	16.2	2.9	4.6	7.1	12.0	14.2	17.3
2	184.9	12.0	2.1	4.5	5.8	7.8	10.1	12.2
3	236.6	13.5	2.4	4.9	7.6	9.1	11.8	13.9
4	377.5	14.4	2.2	4.6	6.7	9.5	11.1	14.5
5	394.0	14.7	3.9	5.2	9.5	12.5	15.0	17.5
6	342.9	20.1	3.2	6.5	9.8	12.3	15.4	17.8
7	359.7	18.3	2.7	4.0	8.7	12.8	14.3	17.0
8	343.3	19.5	2.9	5.5	7.0	9.5	12.4	15.3
9	429.4	20.4	3.2	6.8	9.4	12.3	14.9	17.7
10	249.3	15.0	2.9	5.7	7.4	11.4	13.2	16.6

### 3.3 Multiple brain metastases

**Figure 7:**
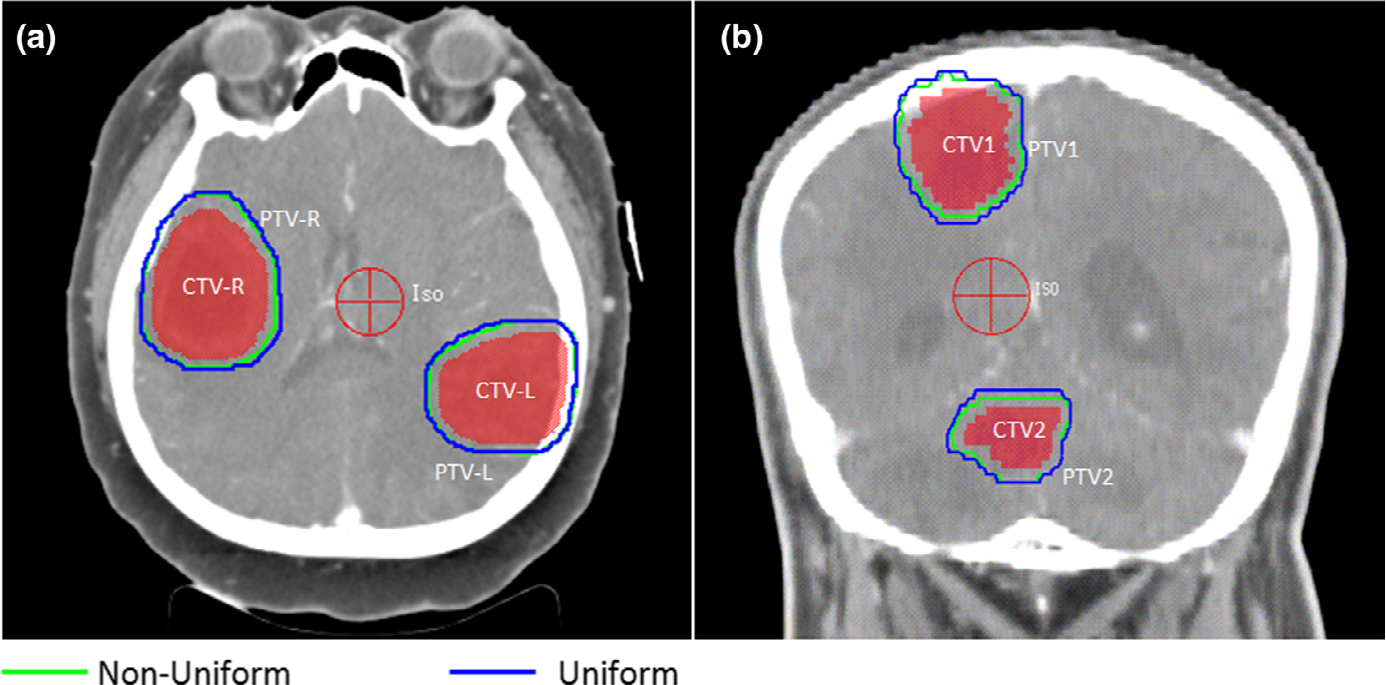
A comparison of planning target volume with two brain metastases targets generated via nonuniform (green) algorithm and uniform (blue) algorithm (a) in transverse view for a patient, (b) in coronal view for another patient. The margin will be small when the corresponding clinical target volume point is close to the isocenter in nonuniform algorithm.

**Figure 8:**
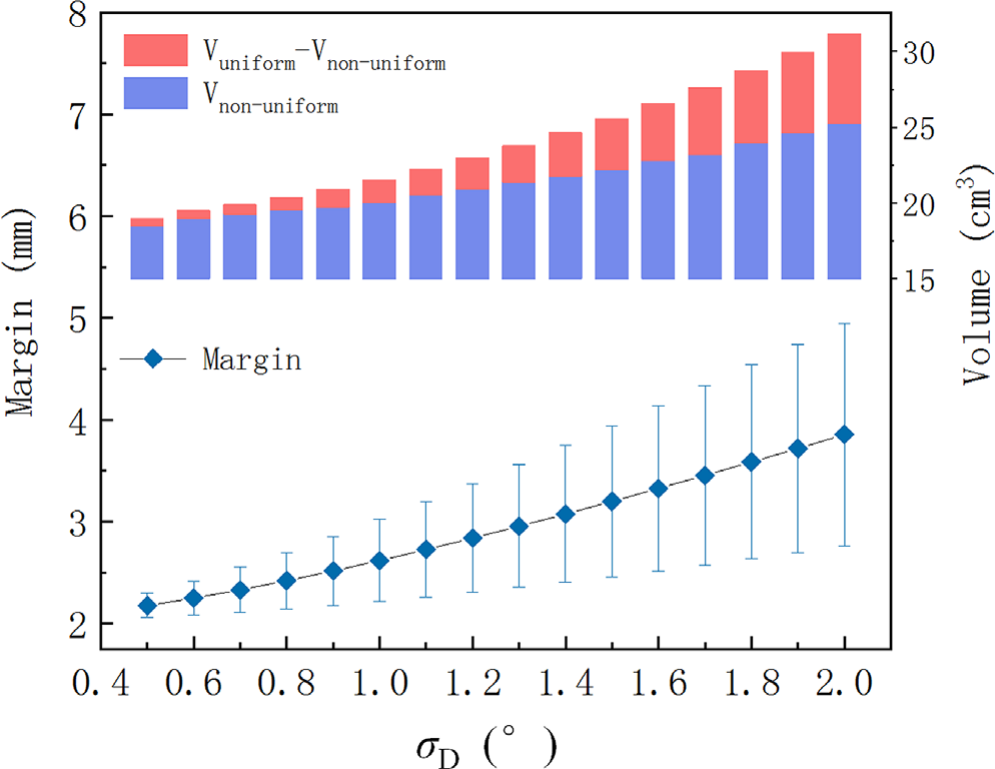
The combined margin and volume of planning target volume1 obtained by nonuniform algorithm versus rotational uncertainty ranging from 0.5° to 2° with 0.1° increment. The nonuniform margin has been drawn as a range bar. Other parameters are $\chi_{\alpha }$ = 2.795, *M*_S_ = 2 mm.

Figure 7 shows comparisons of PTVs generated via nonuniform algorithm (green) and uniform algorithm (blue, *M_E_* = 3.6 mm). As shown in Fig. 7, it is clear that the volume of PTV obtained by nonuniform algorithm is smaller than the PTV volume obtained by uniform algorithm. And the CTV–PTV margin will increase when the target boundary point is moving away from the isocenter. Figure 8 plots the margin and volume of PTV1 obtained by nonuniform algorithm versus rotational uncertainty ranging from 0.5°to 2°with 0.1°increment. From this figure, it is easy to find that the combined margin and volume are positively correlated with rotational uncertainty. The minimum and maximum of the distance between the isocenter and CTV points are 3.1 and 6.4 cm respectively. Table 3 shows the margin and volume of the ten patients’ target treated with SIMT technique.

**Table 3:** The results of the ten patients treated with SIMT technique (M_S_ = 2 mm, *σ*_D_ = 1.0°, $\chi_{\alpha }$ = 2.795).

Patient	Margin_min_ (mm)	Margin_max_ (mm)	V_non‐uniform_ (cm^3^)	V_uniform_ (cm^3^)	Volume difference
1	2.5	3.1	12.9	14.3	9.8%
2	2.3	3.0	12.3	13.5	8.9%
3	2.0	2.4	50.7	57.1	11.2%
4	2.6	3.6	37.2	41.6	10.6%
5	2.3	3.1	103.4	117.1	11.7%
6	2.2	3.4	87.7	97.7	10.2%
7	2.7	3.5	73.5	81.4	9.7%
8	2.4	3.7	81.7	88.6	7.8%
9	2.1	2.9	32.6	36.8	11.4%
10	2.2	3.2	19.3	21.1	8.5%

## 4 DISCUSSION

Based on a statistical model considering both the translational error and the additional rotational uncertainty, we have established a method to obtain a nonuniform CTV–PTV margin based on setup uncertainties. This method was implemented in the Pinnacle^3^ treatment planning system. To prove the advancement of this method, we calculated and compared the volume difference between V_uniform_ and V_non‐uniform_.

From Figs. 4 and 5, it is observed that the combined CTV–PTV margin is related with the distance *d* (between isocenter and CTV point), rotational uncertainty (*σ*_D_), and initial translational margin (*M*_S_). The expansion margin is proportional to the distance between the CTV boundary and the isocenter. For the same rotational uncertainty, the total margin with a smaller initial translational margin will change faster when distance (*d*) changes the same value. So this rotational margin is more important for treatments that require higher precision like the SRS or SBRT, in which PTV margin is usually 2 mm or less. As illustrated in Fig. 4, it is clear that the curves for various *M*_S_ are only a linear function when there is no translational error (*M*_S_ = 0).

For a single target, it can be thought as a combination of many small volume points. Based on the above statistical model, the margin of each point could be obtained. For the rotational margin of each point is different, it will result in a large PTV if we use uniform margin. Because the maximum (near‐maximum) rotational margin of the CTV boundary point is usually chosen as the whole CTV rotational error margin in order to make a safe significance level. Figure 6 shows an example of a vertebral metastases target treated with conventional fractionation. The margins are different for target points at different location. From Table 2, we can see that the volume difference of PTV could be more than 17% when *σ*_D_ value is 1.4°.

For a modern IGRT program equipped with kV on‐board imaging device and six‐degree couch, the residual rotational is generally about 0.5°. Although the volume difference of PTV could be ignored (2%–5%) when translational margin is 2 mm and rotational uncertainty is 0.5°, the result is still meaningful for some special tumor (e.g., multiple metastases). Due to relative motion between various parts of the multiple metastases (vertebral) targets, the uncertainties are impossible to be corrected as expected using six‐degree couch with CBCT. Moreover, new linacs are still being installed with standard couches. The rotational uncertainties will be bigger than 1°. For these patients, the use of a nonuniform margin will make a smaller PTV which may reduce the dose of risk organs nearby. And using a more accurate margin for each part of the target instead of a uniform margin should be the direction of our future efforts for precise radiotherapy.

As shown in Figs. 6, 7 and 8, excessive compensation for the CTV points near isocenter could be avoided in nonuniform algorithm. This method is particularly suitable for the irregular target and multiple targets with single isocenter in which there is a significant difference in the distance from the isocenter to the target boundary points. The irregular target usually has significant differences in shape symmetry. For example, the esophageal tumors or vertebral metastases are usually very long in superior–inferior direction, and are shorter in other directions. Also the location of isocenter is a key issue, and it should be located at the geometric center of CTV in order to obtain the smallest PTV.

The volume of PTV obtained by the nonuniform algorithm is related to the value *d*. On one hand, for a regular target in which the distance from isocenter to target boundary points is almost the same, the rotational margin differs little when the treatment center is at the geometric center of the target area. Therefore, the target volume obtained by these two methods is almost the same. On the other hand, when the volume of the target is so small that the difference between the maximum and minimum values of *d* is negligible, nonuniform algorithm is also not necessary. With regard to specific standards, each institution can determine according to their actual situation.

It should be noted that we focused on the relationship between a nonuniform margin with CTV shape and setup uncertainty in this study The influence of other factors on the target margin is beyond the scope of this paper. But these factors (e.g., respiratory movement, special position fixing device) should be considered when determining the final personal margin. Although a probability of 95% was used in our study, it should be evident that other values can be used without alterations of the algorithm, maintaining the advantages of minimizing the PTV volume for a given probability. From Eq. (1), it is clear that the PTV volume and significance level $\chi_{\alpha }$ are proportional growth relationships. Further research will be carried out to investigate the relationship between reduction of PTV volume with CTV shape, and the position of isocenter. This work was completed with in‐house developed programs, and some features were implemented outside the TPS. More convenient operation design should be completed by the TPS manufacturers.

## 5 CONCLUSIONS

In this paper, we proposed a method to obtain a nonuniform CTV–PTV margin caused by setup uncertainties. This method is based on a statistical model considering both the conventional translational error and the additional rotational uncertainty. The method could avoided excessive compensation for the CTV points near isocenter and was implemented in the clinical treatment planning system (Philips, Pinnacle^3^ 9.10). Compared with the uniform margin algorithm, the advantage of this method is that it will minimize the volume of the PTVs for the given CTVs to obtain the same significance level. This method could be used for clinical margin determination and might be useful for the protection of risk organs nearby.

## CONFLICT OF INTEREST

The authors declare no conflict of interests.
